# Beyond the Ladder: A 10‐Domain Cognitive and Operational Toolkit to Humanise and Implement Acute Pain Guidelines at the Bedside

**DOI:** 10.1155/prm/9154388

**Published:** 2026-10-21

**Authors:** Michele Domenico Spampinato, Sossio Serra, Alessandro Riccardi, Mario Guarino, Claudia Sara Cimmino, Enrico Gandolfo, Fabio De Iaco

**Affiliations:** ^1^ National Study Group on Pain Management and Procedural Sedation and Analgesia (SAU Group) Italian Society of Emergency Medicine (SIMEU) Turin Italy; ^2^ Department of Translational Medicine University of Ferrara Ferrara Italy unife.it; ^3^ Emergency Department Ferrara University Hospital Ferrara Italy unife.it; ^4^ Emergency Department Ramazzini Hospital Carpi Italy; ^5^ Emergency Department Santa Corona Hospital Pietra Ligure Italy; ^6^ Emergency Department AORN dei Colli CTO Hospital Naples Italy unito.it; ^7^ Emergency Medicine Unit 3 ASL Città di Torino Martini Hospital Turin Italy aslcagliari.it; ^8^ Emergency Department ASL Citta di Torino Maria Vittoria Hospital Turin Italy aslto2.piemonte.it

**Keywords:** decision-making, emergency medicine, multimodal analgesia, pain management, palliative care

## Abstract

**Background:**

Although pain is among the most common reasons for emergency department (ED) attendance, oligoanalgesia remains a persistent global issue. While the 2026 EUSEM guidelines provide evidence‐based pharmacological recommendations, translating these into the crowded ED environment is often constrained by systemic overcrowding and a traditional reliance on technical skills, with limited integration of human factors and nontechnical skills relevant to suffering. The objective of this position paper is to propose a practical, human‐centred operational framework that bridges scientific guidelines with clinical realities.

**Discussion:**

The resulting 10‐domain cognitive and operational toolkit synthesises technical and nontechnical skills without implying a mandatory sequence. The domains comprise: (1) establishing psychological safety and therapeutic engagement; (2) multidimensional assessment, including potential indicators of pain and selective neuropathic screening; (3) shared goals and care planning; (4) context‐triggered caregiver integration; (5) severity‐matched, mechanism‐informed multimodal analgesia; (6) individualised opioid stewardship for opioid exposure, tolerance and Opioid Use Disorder; (7) an independent palliative and end‐of‐life domain, including proportional palliative sedation only for appropriately assessed refractory suffering; (8) contextual assessment of broader palliative and social needs; (9) anticipatory workflow, reassessment and rescue planning and (10) handoff and continuity of care. Domains 1, 2, 3, 5, 9 and 10 are core; domains 4, 6, 7 and 8 are context‐triggered. All 10 are briefly considered, while specific tools and actions are activated only when clinically relevant.

**Perspective:**

This position paper addresses the persistent gap between evidence‐based guidelines and the operational realities of overcrowded EDs. By integrating pharmacology with essential nontechnical skills, the proposed 10‐domain toolkit provides a human‐centred cognitive and operational model for daily clinical practice. It is nonsequential: all domains are briefly considered, while core and context‐triggered actions are activated according to clinical relevance. The model requires local adaptation and prospective evaluation.

**Conclusions:**

Effective pain management is an ethical imperative that requires both pharmacological competence and nontechnical skills. This framework serves as a cultural, cognitive and operational guide, helping clinicians embed proactive, individualised pain relief, reassessment and continuity into the demanding workflow of the modern ED. As an expert‐informed toolkit rather than a validated pathway, it requires local adaptation and prospective evaluation.

## 1. Introduction

Although pain remains the most common reason for attending emergency departments (EDs), advances in multimodal therapies and technology, as well as decades of awareness and guideline development, mean the phenomenon of “oligoanalgesia” remains a major, critical challenge in EDs worldwide [1, 2]. Despite guidelines correctly identifying pain as a “sentinel symptom” crucial for diagnosis [3], this diagnostic utility must be balanced against the ethical imperative to treat pain promptly. As established by Italian Law 38/2010 and international declarations, access to pain relief is a fundamental human right and untreated pain constitutes unnecessary suffering that must be addressed without delay [4, 5]. However, in the multicentre study by Todd et al., 60% of patients received analgesia, the median time to administration was 90 min and 74% were discharged with moderate‐to‐severe pain [6].

The release of the 2026 EUSEM Guidelines for the Management of Acute Pain, based on a rigorous systematic review of randomised controlled trials (RCTs), represents a significant and welcome milestone for emergency medicine, aiming to promote a more effective, compassionate and individualised approach [7]. However, translating guidelines into clinical practice remains a complex challenge. While its methodological rigour supports scientific validity, it may inadvertently exclude valuable clinical tools rooted in “human factors engineering” which, although not always validated by large‐scale RCTs, are essential for managing the complexity of human suffering in the chaotic ED environment [8]. High workload and bureaucratic restrictions, viewing pain management as a low clinical priority, failure to recognise the need for improvement in pain management, reliance on experience rather than the application of new knowledge and lack of staff trust in patients’ ability to judge pain appropriately are acknowledged barriers to effective pain management [9]. Recently, a Failure Modes, Effects and Criticality Analysis (FMECA) of the pain journey in the ED identified communication, decision support, protocolisation and pain education as key failure factors in effective pain management [10]. This suggests that publishing guidelines alone is not sufficient: we need to refine how these recommendations are implemented in clinical practice. Addressing oligoanalgesia requires a dual approach: evidence‐based multimodal pharmacology [7, 11, 12], and implementation strategies that address communication failures, staff education, decision support and workflow integration [9, 13].

In this narrative position paper, we propose a 10‐domain operational framework intended to offer a systematic, human‐centred approach for translating evidence‐based acute pain recommendations into emergency care practice. The framework is presented as an implementation aid and educational structure, not as a mandatory sequence, a formally developed clinical practice guideline or an externally validated consensus statement.

### 1.1. Approach, Scope and Evidence Base

This article is a narrative position paper based on a critical, nonsystematic review of the literature and on the authors’ clinical and educational experience in acute pain management within emergency medicine. It does not present original empirical data, does not report implementation outcomes, and was not developed through a Delphi process, a GRADE‐based evidence‐to‐decision process, a formal hierarchy of evidence, an international multidisciplinary panel or external validation. Consequently, the statements below should be read as expert‐informed proposals that require local adaptation and prospective testing rather than as prescriptive recommendations.

#### 1.1.1. Literature Search Strategy

To support the perspectives presented here, we conducted a narrative literature review focused on acute pain management, analgesic stewardship, human factors and emergency care implementation. PubMed, Scopus and Web of Science were searched from inception to March 2026; however, no formal systematic‐review protocol, duplicate screening, risk‐of‐bias assessment or meta‐analysis was undertaken.

We prioritised peer‐reviewed original studies, systematic reviews, internationally recognised guidelines and implementation reports that were considered most relevant to ED pain pathways. The selection of sources was pragmatic and explanatory rather than exhaustive.

#### 1.1.2. Integration of Expertise and Internal Agreement

The theoretical and practical positions outlined in this article stem from the authors’ direct clinical experience in managing patients with pain in emergency and urgent care settings and from scientific and educational activities within the Italian Society of Emergency Medicine (SIMEU), including more than 50 training courses delivered nationwide over the past decade. The framework was formalised rather than generated de novo for this manuscript: its domains reflect recurring clinical problems, decision points and practical tools that emerged through these activities and were repeatedly discussed in clinical teaching.

For the present manuscript, these recurring elements were organised and refined through an iterative process:

- Clinical‐educational scoping: identifying recurrent problems, decision points and practical tools in emergency pain care based on the authors’ clinical experience and SIMEU educational activities.
- Literature cross‐validation: comparing each proposed domain and tool with the empirical evidence, guidelines and implementation literature identified in the narrative review and revising claims where the evidence did not support a stronger formulation.
- Internal agreement: discussing each domain and proposed tool among all authors until a shared formulation was reached. Disagreements were resolved through iterative discussion rather than formal voting or numerical scoring.

No formal candidate‐item pool, ranking exercise, quantitative scoring system or predefined inclusion threshold was used. The resulting 10 domains therefore reflect the iterative formalisation of an existing clinical‐educational model rather than a formal consensus methodology.

The domains were retained because, in combination, they captured recurrent clinical and implementation issues across the ED pain‐care trajectory. Their numerical labels are used solely for reference and do not imply sequence, priority or mandatory progression.

To make the provenance of each component explicit, Table 1 distinguishes established or validated practices and instruments, adaptations from other clinical contexts and author‐derived educational or implementation heuristics. These descriptors are not formal evidence grades. The table also identifies each domain’s intended context, action class and principal supporting evidence.

**TABLE 1:** Evidence provenance, intended clinical context and action class of the proposed 10‐domain toolkit.

Domain	Action class	Evidence provenance/validation status	Intended clinical context and principal supporting evidence
1. Psychological safety and therapeutic engagement	Core	Concept supported by pain‐threat and communication literature; AIDET is an adapted communication scaffold whose use in ED acute‐pain care has not been specifically validated.	Early encounter; reduce uncertainty and support communication [14, 15].
2. Multidimensional assessment	Core; selected subtools are context‐triggered	Population‐specific pain scales and DN4 are validated instruments. Physiological and behavioural signs are potential, nonspecific indicators rather than diagnostic “pain equivalents”.	All patients; behavioural tools when self‐report is unreliable; DN4 when neuropathic/mixed features are suspected [16–18].
3. Shared goals and care plan	Core	Shared decision‐making is established practice; the acute‐pain use of SPIKES is an adapted, unvalidated communication scaffold.	Set realistic, individualised and function‐oriented goals; agree on reassessment and rescue [19–21].
4. Caregiver integration	Context‐triggered	Social support is supported by broader evidence. The proposed caregiver observation prompt is adapted from CPOT domains and is not a validated family‐administered CPOT.	When the patient cannot self‐report or the caregiver can add meaningful contextual/behavioural information, subject to consent, privacy and workflow [22, 23].
5. Severity‐matched, mechanism‐informed multimodal analgesia	Core	Severity‐matched, mechanism‐informed multimodal analgesia and selected non‐pharmacological/regional techniques are established practices. CERTA is a published educational mnemonic proposed by Cisewski and Motov [24] and adopted in the 2026 EUSEM guideline. ODO is an author‐derived educational heuristic. Neither is a validated clinical decision rule.	Match treatment intensity, mechanism, onset/duration and monitoring to clinical need; reassess and de‐escalate [7, 24].
6. Opioid exposure, tolerance and OUD	Context‐triggered	Opioid stewardship and equianalgesic conversion principles are evidence‐informed; MME/conversion tables are approximate estimates, not mathematically precise rules.	Opioid‐tolerant or opioid‐exposed patients and OUD: verify baseline treatment; individualise conversion and rescue dosing; continue established MOUD when appropriate; use local protocols for OUD initiation or major modification [25–27].
7. Palliative and end‐of‐life needs	Context‐triggered	Palliative sedation is addressed by contemporary palliative‐care frameworks and is reserved for appropriately assessed refractory suffering.	Advanced illness/active dying or suspected refractory suffering: goals of care, reversible contributors, symptom treatment, refractoriness, consultation when available, and proportional sedation only when indicated [28, 29].
8. Broader palliative and social context	Context‐triggered	The need for ED palliative screening is supported by systematic‐review literature; 5‐SPEED is a previously described five‐question adaptation of the 13‐item SPEED instrument, for which content validity and reliability have been reported. It has no validated numerical score or threshold for general ED use, and its extension beyond oncological populations requires prospective validation.	When pain may signal broader deterioration, social fragility, access problems or unmet palliative needs [30, 31].
9. Anticipatory workflow, reassessment and rescue	Core	Nurse‐initiated analgesia is supported by systematic‐review evidence; scheduled non‐opioid baseline and rescue planning are implementation tactics requiring local protocols.	Reduce crowding‐related delay, plan reassessment and rescue, and pursue meaningful symptom/function improvement [32].
10. Handoff and continuity	Core	Continuity and opioid stewardship are established principles; the specific receiving clinician depends on the healthcare system.	All dispositions: ED recognition and initial mechanism‐informed treatment, written plan, red flags, reassessment, responsible follow‐up and pain, palliative or social linkage when needed [25, 31].

*Note:* “Core” and “context‐triggered” describe how actions are activated. All 10 domains are briefly considered, but only clinically relevant instruments and interventions are used. The evidence‐provenance categories are descriptive rather than formal evidence grades.

### 1.2. Proposed Framework: The 10‐Domain Cognitive and Operational Toolkit

To bridge the implementation gap, we propose a modular 10‐domain toolkit integrating human factors, pain phenotyping, pharmacology, workflow and continuity. It is neither a sequence nor a ten‐item bedside checklist. All domains are briefly considered as a safeguard against overlooking clinically relevant dimensions of pain and suffering; specific instruments and interventions are used only when appropriate. Core actions (therapeutic engagement, multidimensional assessment, shared goals, severity‐matched, mechanism‐informed multimodal analgesia, reassessment and rescue planning and continuity) are usually relevant to most patients. Caregiver involvement, opioid exposure, tolerance or opioid use disorder (OUD), palliative and end‐of‐life needs and broader palliative or social complexity are context‐triggered. This structure preserves the emergency physician’s comprehensive responsibility while remaining feasible in crowded EDs. Departments should adapt, pilot and audit the toolkit according to staffing, protocols and patient mix (Table 1; Figure 1).

**FIGURE 1:**
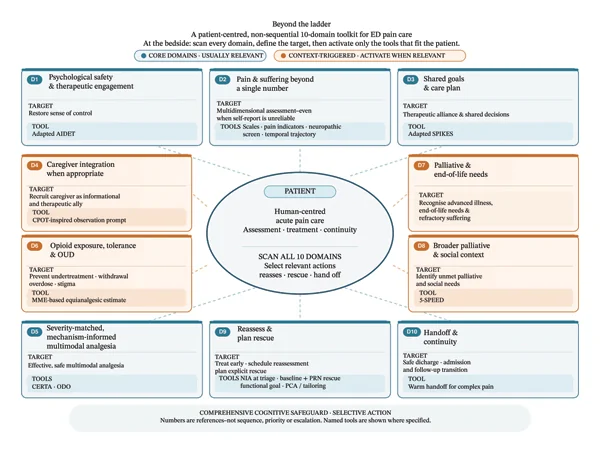
Patient‐centred, nonsequential representation of the 10‐domain cognitive and operational toolkit for acute pain and suffering in the emergency department. Six core domains are usually relevant to most patients; four context‐triggered domains are activated when clinically appropriate. All domains are briefly considered. Numbering is for reference only and does not indicate a mandatory sequence, priority or escalation. Abbreviations: ED, emergency department; OUD, opioid use disorder.

### 1.3. Domain 1: Establish Psychological Safety and Therapeutic Engagement

#### 1.3.1. Target: Restore the Sense of Control

In the acute setting, unmet basal needs tend to dominate the patient’s experience: Maslow’s Hierarchy of Needs offers a useful conceptual framework here, whereby physiological needs (pain relief) and safety needs (protection from danger) take precedence over higher‐order needs when threatened [33–35]. Fear and uncertainty may amplify pain through threat‐related cognitive and affective processes; reducing uncertainty and restoring a sense of safety are therefore early components of comprehensive pain care [14, 36]. Simultaneously, minimising sensory overload (by reducing noise and dimming lights) may reduce the arousal state that exacerbates pain processing [37, 38].

#### 1.3.2. The Suggested Tool: The Adapted AIDET “Acknowledge, Introduce, Duration, Explanation and Thank You” Framework

The AIDET framework offers a simple structure for acknowledging the patient, introducing oneself, clarifying expected timing and next steps and explaining procedures. Originally developed outside ED acute‐pain care, it is used here as an adapted communication scaffold rather than a validated analgesic intervention. By reducing uncertainty and strengthening trust, it may help address anxiety‐related amplification of pain, although a direct analgesic effect or specific amygdala‐mediated mechanism has not been established [15].

### 1.4. Domain 2: Assess Pain and Suffering Beyond a Single Number

#### 1.4.1. Target: Multidimensional Assessment, Including Potential Indicators of Pain When Self‐Report Is Unreliable

Pain, nociception and suffering are related but distinct. Pain is a subjective sensory and emotional experience; nociception refers to the neural processing of noxious stimuli; and suffering encompasses broader physical, emotional, social or existential distress [39]. Distinguishing them is clinically important, especially when self‐report is limited. In patients able to communicate, self‐report remains the reference standard. In noncommunicative, paediatric or cognitively impaired patients, inability to verbalise pain does not exclude it and assessment should combine population‐specific validated tools, clinical context and observed behaviour.

#### 1.4.2. The Suggested Tools: Validated Scales, Potential Indicators of Pain, Selective Neuropathic Screening and Temporal Trajectory

One size does not fit all: the use of the correct age‐appropriate and cognitive‐specific validated tool is a prerequisite for effective treatment.

• Adults (Competent): Numeric Rating Scale (NRS) or Visual Analogue Scale (VAS) [16].
• Paediatrics (Verbal): Faces Pain Scale–Revised (FPS‐R) or Wong–Baker FACES Scale [17].
• Paediatrics (Nonverbal/Toddlers): Face, Legs, Activity, Cry, Consolability (FLACC Scale) [17].
• Neonates/Infants: Specific validated tools based on gestational age, such as Premature Infant Pain Profile (PIPP) or Neonatal Facial Coding System (NFCS) [17, 40].
• Elderly individuals with cognitive impairment or dementia can be assessed using the Pain Assessment in Advanced Dementia (PAINAD) tool [41].
• Noncommunicative/Critical Care Adults: Behavioural Pain Scale (BPS) or Critical‐Care Pain Observation Tool (CPOT) [16].

In patients who cannot reliably self‐report, otherwise unexplained physiological or behavioural distress should prompt active consideration of pain while life‐threatening and reversible alternative causes are assessed in parallel. These signs are potential indicators of pain, not diagnostic equivalents.

• Potential physiological indicators: tachypnoea, tachycardia, hypertension or diaphoresis may accompany uncontrolled nociception but are nonspecific and should be interpreted alongside alternative causes such as hypoxaemia, infection, shock, withdrawal, intoxication, metabolic disturbance or medication effects.
• Potential behavioural indicators: agitation, delirium, combativeness, guarding or altered behaviour may accompany pain, particularly when self‐report is unreliable. They should prompt parallel assessment for neurological, medical, toxicological and psychiatric causes rather than an assumption that pain or hypoxaemia is primary [42, 43].

Standard intensity scales do not characterise pain phenotype. Because recognising a neuropathic component may alter acute treatment and disposition, emergency clinicians should actively consider neuropathic features during assessment [44]. DN4 is a brief validated tool that can be used when history or examination suggests neuropathic or mixed pain [18], rather than as a mandatory screen in every ED patient. Recognition should guide mechanism‐informed treatment of the acute episode and appropriate follow‐up.

Pain is not simply a momentary sensation: its trajectory and interference with function can inform both diagnosis and treatment. In persistent, recurrent or complex pain, when clinically relevant, structured questions or tools such as the Brief Pain Inventory (BPI) may help explore worst/least pain, function, mood, sleep and movement over time [45]. Understanding whether pain is stable, escalating or fluctuating may also reveal a trajectory that a single triage score cannot capture.

### 1.5. Domain 3: Establish Shared Goals and a Care Plan

#### 1.5.1. Target: Therapeutic Alliance and Shared Decision‐Making

Analgesia is not simply the unilateral delivery of drugs; it also requires a therapeutic alliance and shared expectations. The Setting, Perception, Invitation, Knowledge, Emotion and Strategy (SPIKES) protocol provides a useful framework for difficult conversations [19]. We adapt it here as an educational scaffold for acute‐pain conversations—to elicit fears and expectations, share information and agree on goals—while recognising that this use has not been specifically validated in the ED [20, 21].

#### 1.5.2. The Suggested Tool: The Adapted SPIKES Model for Acute Pain Management

• S (Setting): Ensure privacy (even within a curtained bay) and, if possible, sit down to signal presence.
• P (Perception): Ask, “What do you think is causing this pain?” to identify fears or misconceptions.
• I (Invitation): “Is it okay if we discuss a plan to manage your suffering together?”
• K (Knowledge): Manage expectations explicitly. Example: “We will keep reassessing your pain and adjust the treatment according to your response and safety. Our immediate goal is a meaningful reduction that allows you to breathe, move or rest as safely as possible; complete elimination of pain may not always be immediately achievable or desirable.”
• E (Emotion): Validate the distress. “I can see how exhausting this situation is for you.” Validation may reduce distress and support the therapeutic alliance.
• S (Strategy): Agree on the plan, including the first intervention, the expected time to reassessment and the rescue or adjustment strategy if the initial response is inadequate.

### 1.6. Domain 4: Integrate Caregivers When Clinically Appropriate

#### 1.6.1. Target: Context‐Triggered Recruitment of the Caregiver as a Therapeutic and Informational Ally

While family presence is standard in paediatric care, adult EDs may still treat caregivers primarily as visitors. When safety, consent, privacy and workflow allow, structured caregiver involvement can be considered as an adjunct to pain assessment and reassurance. Social support may reduce distress and improve coping [22], but it should be applied selectively rather than as a universal mandate, particularly during invasive procedures or resuscitation [46].

#### 1.6.2. The Suggested Tool: A Caregiver Behavioural Observation Prompt Inspired by CPOT Domains

To involve the caregivers as a supportive observer when appropriate, we propose a brief educational script inspired by observable behavioural pain principles, included in the CPOT domains (facial expression, body movements, muscle tension, vocalisation) [23, 47]. This is not a validated family‐administered CPOT; it is an optional communication technique designed to focus relatives on observable behaviours and reduce helplessness. Family members may recognise subtle changes in expression or behaviour that are not captured during intermittent staff observations. Their input can therefore complement, but not replace, validated pain assessment and clinical judgement.

• Validate their expertise: “You know his expressions better than anyone. We need you to interpret them for us.”
• Assign Specific Markers: “Focus on two things: his face and his hands. If you see him frowning or grimacing (facial expression), or if his hands feel tight and he is resisting movement (muscle tension), that is our signal.”
• Close the loop: “If you observe these changes, please alert us.” Focusing attention on observable behaviour may reduce caregiver helplessness and add contextual information between staff assessments, although all observations remain nonspecific and require clinical interpretation.

### 1.7. Domain 5: Use Severity‐Matched, Mechanism‐Informed Multimodal Analgesia

#### 1.7.1. Target: Effective and Safe Multimodal Analgesia

Consistent with the 2026 EUSEM guideline, the toolkit combines nonpharmacological measures with mechanism‐informed multimodal pharmacotherapy. Alongside pharmacotherapy, emergency physicians should optimise simple nonpharmacological measures when feasible. Positioning, splinting and cooling may provide useful adjunctive analgesia in selected musculoskeletal injuries and should not delay necessary pharmacological treatment [48]. Communication and expectation‐setting can support pain care but should not substitute for indicated therapy. Low‐risk adjuncts such as distraction or music may be considered in selected patients; emerging ED trials suggest possible benefits for pain and anxiety, but these interventions remain adjunctive [49–51].

While the WHO Pain Ladder remains an important educational framework, it was originally developed for cancer pain and should not be applied mechanistically to all acute ED presentations [28, 52, 53]. For severe acute pain, we favour severity‐matched, mechanism‐informed multimodal analgesia initiated without requiring progression through weaker therapies when stronger treatment is clinically indicated, followed by prompt reassessment, titration and de‐escalation as the cause is treated. This approach is intended to avoid reflexive step‐up delay, not to mandate routine pharmacological escalation. The specific regimen should follow evidence‐based recommendations, patient factors, monitoring capacity and local protocols.

#### 1.7.2. The Suggested Tools: Channels‐Enzymes‐Receptors Targeted Analgesia (CERTA) and Onset‐Duration‐Offset (ODO) Heuristics

To structure effective and safe multimodal pain management, we propose the use of two heuristics:

1. CERTA: an educational mnemonic proposed by Cisewski and Motov [24] and subsequently adopted as an organising framework in the updated EUSEM guideline for acute pain management [7]. It is intended to shift attention from drug classes toward mechanism‐informed multimodal analgesia and should not be interpreted as a validated clinical decision rule.
  • Peripheral Transduction (e.g., NSAIDs and local anaesthetics).
  • Ascending Transmission (e.g., sodium channel blockers and opioids).
  • Central Processing and Descending Inhibition: This stage is frequently overlooked. Selected patients may benefit from agents that modulate central elaboration of pain, such as subdissociative ketamine or alpha‐2 agonists, when supported by local protocols, monitoring capacity and clinician expertise.
2. Author‐derived educational heuristic: ODO. We propose ODO as an author‐derived educational mnemonic: a pharmacokinetic prompt for considering how drug timing relates to the anticipated pain trajectory, rather than a validated decision rule. To our knowledge, this acronym has not previously been described as a named framework in the analgesia or emergency medicine literature; the individual components it groups, however, are well‐established pharmacokinetic constructs [54, 55]. The mnemonic is intended solely to structure teaching and bedside reasoning and should not be interpreted as a validated protocol or a substitute for individualised clinical judgement.
  • O (Onset): Consider the time to clinically meaningful effect and match it to clinical urgency. Hyperacute pain may justify a rapid‐onset agent, whereas less urgent settings may accommodate agents with a slower onset; relative onset differs markedly between opioids [54]. Agent selection should also account for patient factors, adverse‐effect profile, route and monitoring capacity [56].
  • D (Duration): Match the expected duration of clinical effect to the anticipated temporal trajectory of the pain source [54]. A mismatch may expose the patient either to breakthrough pain when the effect is too short or to cumulative toxicity and delayed adverse effects when drug action substantially outlasts clinical need.
  • O (Offset and bridging): Consider redistribution, metabolism and context‐sensitive offset when planning the next phase of therapy [57, 58]. Starting maintenance analgesia before the initial effect has completely dissipated may reduce gaps in coverage and breakthrough pain. This anticipatory approach should be guided by drug‐specific pharmacokinetics, clinical response and monitoring; however, evidence that it prevents central sensitisation or pain chronification in emergency‐department populations is lacking.

The practical consequence is to design an analgesic plan that anticipates loading, maintenance, overlap timing and rescue provision while preserving reassessment and de‐escalation.

Regional anaesthesia is an important opioid‐sparing option in selected ED patients when trained clinicians, ultrasound equipment, monitoring and local governance are available. ACEP and EUSEM educational frameworks recognise ultrasound‐guided nerve blocks as a relevant emergency medicine skills [59–61]. Fascial plane blocks (e.g., Erector Spinae, Fascia Iliaca and Serratus Anterior plane blocks) may offer favourable safety and accessibility profiles compared with deeper plexus techniques, but they require training, audit and adherence to local safety protocols [62, 63].

### 1.8. Domain 6: Recognise Opioid Exposure, Tolerance and OUD

#### 1.8.1. Target: Avoiding Undertreatment, Withdrawal, Overdose and Stigma Through Individualised Opioid Stewardship

Managing severe pain in patients receiving long‐term opioids for chronic or cancer‐related pain, and in those with OUD, remains challenging. These patients are vulnerable both to undertreatment because of tolerance and stigma and to harm from unsafe dose escalation. Before escalating or discharging with opioids, assess pain phenotype, previous opioid exposure, respiratory risk, renal/hepatic function, sedative co‐medication, frailty, pregnancy where relevant, substance‐use history and local prescription‐monitoring data where available. Use shared decision‐making to explain expected benefits, adverse effects, safe storage and disposal and arrange follow‐up rather than relying on the label of opioid‐seeking behaviour as a clinical shortcut [25]. The clinical task is therefore not to deny opioids categorically nor to prescribe them reflexively, but to combine adequate analgesia with explicit opioid stewardship.

#### 1.8.2. The Suggested Tool: Equianalgesic Estimates via MME Calculation

MME calculations and equianalgesic tables provide approximate clinical estimates rather than mathematically precise equivalence. In opioid‐tolerant patients, emergency physicians should estimate the opioid actually taken over the preceding 24 h, preferably using a locally validated converter or pharmacist support, and consider the specific opioid and route, prior exposure, incomplete cross‐tolerance, age, frailty, renal or hepatic function, sedative co‐medication and clinical context [26, 64]. For patients with established tolerance and a verified stable regimen, a short‐acting rescue dose for breakthrough pain is often initially estimated at approximately 10%–15% of the effective 24‐h opioid dose; this value is a starting range, not a fixed rule, and requires route‐specific adjustment, close reassessment and titration. During opioid rotation, the calculated equianalgesic dose is commonly reduced by approximately 25%–50% to account for incomplete cross‐tolerance, with the reduction individualised according to pain severity and patient risk before titration to effect. Methadone, transdermal fentanyl, very high doses and unstable organ function require particular caution and should be managed according to local protocols, with pharmacist or pain/addiction specialist support when available.

Patients with OUD remain vulnerable to undertreatment when pain reports are discounted or behaviour is labelled as opioid‐seeking. Acute analgesia should therefore be delivered with the same trauma‐informed, nonstigmatising assessment applied to other patients, while distinguishing pain treatment from the initiation or major modification of medication for OUD (MOUD). For patients already receiving MOUD, the verified maintenance dose of buprenorphine or methadone should generally be continued; maintenance methadone alone should not be assumed to provide adequate acute analgesia. The total daily buprenorphine dose may be divided into three or four administrations during the acute‐pain period to improve analgesic coverage, preferably in coordination with the usual prescriber. Multimodal nonopioid and regional strategies should be maximised; moderate‐to‐severe pain may require additional short‐acting full μ‐opioid agonists at opioid‐tolerant doses, with fentanyl or hydromorphone commonly favoured in buprenorphine‐treated patients and titrated under close respiratory monitoring. These recommendations concern analgesia in patients already receiving MOUD. Initiating or substantially modifying buprenorphine or methadone for OUD should follow local protocols and established linkage pathways [27, 65].

### 1.9. Domain 7: Recognise Palliative and End‐of‐Life Needs

#### 1.9.1. Target: Advanced Illness, End‐of‐Life Care and Appropriately Assessed Refractory Suffering

Standard acute‐pain guidelines typically address reversible conditions and offer limited guidance when a patient is actively dying or experiencing severe suffering despite comprehensive, interprofessional palliative care that does not compromise consciousness [29, 66]. Pain and other sources of distress still require active management at the end of life [28]. In this setting, however, continuing to escalate opioids may be ineffective or harmful, and inadequate analgesia alone does not establish an indication for sedation. Goals of care and decision‐making preferences should be clarified, potentially reversible contributors addressed and symptom‐specific treatment optimised before suffering is deemed refractory. Only then, when refractory suffering is established, may Proportional Palliative Sedation (PPS) be appropriate, with depth and dose guided by proportionality, informed decision‐making, local policy and the applicable legal framework [28, 29, 67]. PPS is therefore considered within an independent, context‐triggered palliative and end‐of‐life domain, rather than as an escalation after opioid therapy. PPS must be clearly and rigorously distinguished from euthanasia [67]. As established by the European Society for Medical Oncology (ESMO), the European Association for Palliative Care (EAPC) and extensive literature, the distinction lies in the intent and the procedure [68–70]:

• Intent: The goal of PPS is relief of appropriately assessed refractory suffering, not the hastening of death [71].
• Proportionality and titration: Sedation should be titrated to the minimum level required to relieve refractory suffering, with depth and dose proportional to the clinical need and goals of care [29, 72].
• Outcome: PPS is intended to relieve otherwise refractory suffering. Current evidence does not demonstrate that appropriately indicated and correctly administered palliative sedation shortens survival, although no randomised trials exist and the available data reflect an absence of evidence for a life‐shortening effect rather than proof of its absence [73, 74]. Its success should nevertheless be judged by relief of suffering, proportionality and concordance with patient goals rather than by an assumed “quality of death” [72].

The principle of double effect has historically informed end‐of‐life symptom treatment when benefit is intended and harm is foreseeable but unintended. Contemporary justification for PPS is broader and rests on refractory suffering, proportionality, patient goals and consent, professional standards and applicable law. Neuropalliative guidance likewise supports adequate treatment of pain, dyspnoea, anxiety and other sources of acute discomfort in dying patients while recognising foreseeable treatment risks [71].

Legal frameworks vary by jurisdiction. In Italy, Law 219/2017 provides an explicit legal framework for deep continuous palliative sedation in association with pain therapy in the presence of refractory suffering, subject to informed consent or surrogate decision‐making [75]. This national example should be interpreted within the applicable legal and institutional framework in other settings.

### 1.10. Domain 8: Unveil the Broader Palliative and Social Context

#### 1.10.1. Target: Identify Broader Unmet Palliative and Social Needs Using a Brief Contextual Prompt

Once immediate threats and urgent symptom control have been addressed, the clinician should broaden the lens when clinically relevant. Acute pain in the ED can be a sentinel event indicating deterioration in a patient’s chronic trajectory. Standard history‐taking may miss social frailty, support systems, access to care and psychological or palliative needs that shape the experience and management of pain.

#### 1.10.2. The Suggested Tool: 5‐Screen for Palliative and End‐of‐life Care Needs in the ED (SPEED)

The SPEED is a 13‐item, content‐valid and reliable tool developed for ED patients with cancer, organised across five domains (physical, therapeutic, psychological, social and spiritual needs) [76]. It was subsequently adapted into a rapid five‐question needs assessment, the 5‐SPEED, which retains one item per key area (pain management, home care, medication management, psychological support and goals of care) [77, 78].

The 5‐SPEED questions [77, 78]:

1. How much are you suffering from pain?
2. How much difficulty are you having getting your care needs met at home?
3. How much difficulty are you having with your medications?
4. How much are you suffering from feeling overwhelmed?
5. How much difficulty are you having getting medical care that fits with your goals?

Taken together, the five questions broaden the assessment from symptom intensity to the physical, psychological and social context in which pain occurs. Concern in any one domain may justify focused evaluation and, when appropriate, early linkage to palliative care, social work, primary or community care or other support. This may help avoid an isolated pharmacological response when pain is embedded in broader unmet needs; effects on repeat ED use and patient‐centred outcomes remain to be evaluated. Despite 5‐SPEED being among the tools appraised in the systematic review of ED palliative‐care screening [30], its predictive validity has not been established outside oncological populations, and this extension should be regarded as a recommendation requiring prospective validation in noncancer ED patients rather than an already proven application [77, 78].

### 1.11. Domain 9: Anticipate, Reassess and Plan Rescue Analgesia

#### 1.11.1. Target: Reduce Crowding‐Related Delay Through Early Treatment, Planned Reassessment and Explicit Rescue Strategies

In the modern ED, overcrowding and prolonged door‐to‐doctor times create an analgesic gap [6]. We propose a pragmatic shift from purely reactive prescribing to anticipatory protocols that remain simple enough for local workflow.

#### 1.11.2. The Suggested Tools

##### 1.11.2.1. Nurse‐Initiated Analgesia (NIA) at Triage

Pain management should begin as early as clinically feasible. Systematic‐review evidence associates NIA understanding protocols with safe, timely and effective pain relief; it does not establish a consistent reduction in overall ED length of stay [32].

##### 1.11.2.2. Scheduled Nonopioid Baseline Versus PRN Opioid Rescue

For ongoing pain, relying exclusively on PRN orders can allow recurrent breakthrough pain before the patient receives help. However, scheduled therapy should not be interpreted as automatic scheduled opioid therapy. We suggest fixed‐schedule dosing for the nonopioid multimodal baseline, such as acetaminophen/paracetamol, NSAIDs when not contraindicated, local/regional techniques and selected adjuvants. Opioids should generally remain PRN rescue medications for moderate‐to‐severe breakthrough pain, given at the lowest effective dose with reassessment and monitoring. Scheduled opioids may be appropriate only for selected patients, such as those under observation/admission, opioid‐tolerant patients requiring a temporary bridge or palliative/end‐of‐life situations, and should include a reassessment and taper/de‐escalation plan [79].

##### 1.11.2.3. The “Functional Goal” Strategy

For ongoing severe pain, the clinician should establish individualised treatment goals before any repeated escalation. The target should shift from a universal expectation of “zero pain” toward meaningful symptom relief and functional improvement, for example, the ability to breathe deeply, mobilise, cooperate with care or rest. Goals should be reassessed as the clinical condition evolves, balancing analgesia with sedation, respiratory and other treatment risks.

##### 1.11.2.4. Patient‐Controlled Analgesia (PCA) and Tailoring

For sustained severe pain in selected monitored patients, intermittent boluses may be inadequate. PCA may improve autonomy and analgesic titration in some settings, but ED evidence remains limited and implementation requires monitoring capacity, nursing protocols and clear escalation criteria [80].

### 1.12. Domain 10: Plan Handoff and Continuity of Care

#### 1.12.1. Target: Safe Transition Across Discharge, Admission and Longitudinal Follow‐Up

The emergency physician’s responsibility extends beyond the exit door. Emergency care should extend beyond immediate symptom control to include a safe transition plan, particularly when pain is complex, chronic, neuropathic, opioid‐related or associated with broader unmet needs. An isolated ED prescription is insufficient without a safety net involving pain specialists, palliative‐care teams, social services and longitudinal care providers when appropriate. Transition planning should be risk‐stratified by pain phenotype, comorbidities, opioid exposure and expected duration of severe pain. The written plan should include nonpharmacological measures, scheduled nonopioid analgesia when appropriate, clearly defined PRN rescue options, red flags, reassessment timing and named follow‐up responsibility. Opioid prescribing at discharge should apply the same principles of mechanism‐informed care and stewardship, with particular emphasis on patient safety.

#### 1.12.2. The Suggested Tool: The “Warm Handoff” for Complex Pain

Recognition and initial treatment of neuropathic, refractory or complex chronic pain are part of emergency care. The ED clinician should identify the likely pain phenotype, address reversible contributors, provide mechanism‐informed treatment for the acute episode and define a safe transition plan. Pain‐service referral complements this care when longitudinal diagnostic review, co‐analgesic titration or interventional management is needed. When opioids are prescribed at discharge for acute‐on‐chronic or complex pain, the quantity should be limited to the shortest clinically appropriate duration according to diagnosis, expected pain trajectory, local regulations and follow‐up availability, with earlier reassessment when risk is high [25].

When the 5‐SPEED prompt or broader clinical assessment identifies unmet palliative or social needs, the priority is targeted further assessment and appropriate linkage to palliative care, social services, home care or other support. Coordinated support may reduce repeat ED use and improve continuity [31]. In this context, any opioid prescription should form part of a broader symptom‐management and follow‐up plan rather than function as an isolated intervention.

For patients discharged with ongoing analgesic needs, the primary care clinician or responsible longitudinal provider, according to the local healthcare system, should be actively informed rather than passively copied whenever feasible. This clinician can reassess pain trajectory, adverse effects, function and any tapering plan. We support a prescribe‐to‐partner approach in which discharge opioid prescribing, when indicated, is linked to a clear follow‐up plan and identified responsibility for reassessment and risk mitigation.

### 1.13. Limitations and Need for Validation

This manuscript has important limitations. It is a narrative position paper rather than a systematic review, formal guideline, Delphi consensus, GRADE‐based recommendation or externally validated care pathway. The 10‐domain architecture and core/context‐triggered distinction reflect expert synthesis. Table 1 distinguishes established or validated components from adaptations and author‐derived heuristics, but no formal item‐generation, ranking or external consensus process was undertaken. Feasibility, acceptability, safety and effects on patient‐centred outcomes require prospective evaluation in diverse ED settings.

Because the framework is intentionally focused on emergency care, its immediate readership may be clinicians and researchers interested in acute pain implementation, emergency medicine and transitional pain stewardship. Its contribution to pain medicine is therefore translational: it identifies operational points at which evidence‐based pain care can fail and proposes targets for future pilot implementation, audit and refinement.

## 2. Discussion

Effective management of acute pain in the ED extends beyond symptom control. It is a clinical and ethical priority that requires pharmacological competence, nonpharmacological measures, communication, reassessment and continuity planning. Practices such as psychological safety, empathetic communication and shared decision‐making should be understood as adjunctive components of care that may modulate suffering and improve adherence, while remaining subject to the same standards of evaluation as other implementation strategies.

The 2026 EUSEM guideline provides evidence‐based recommendations for acute‐pain treatment, while recent ED pathway and implementation studies have identified failure points involving communication, workflow, decision support and knowledge translation [9, 10, 13]. The proposed toolkit complements these approaches through a clinician‐facing cognitive architecture that combines established practices, adapted nontechnical tools and explicitly labelled author‐derived heuristics across the patient journey from first contact to disposition and continuity.

We fully acknowledge the reality of current EDs: overcrowding, staff shortages and boarding are major barriers to personalised pain management. Under these conditions, the 10‐domain toolkit should be implemented as a locally adapted, tiered model rather than as a time‐consuming list. The relevance of all domains is considered, but context‐triggered actions are activated only when clinically indicated. Local needs assessment, staff training, nurse‐initiated protocols and audit of time‐to‐analgesia, patient‐centred outcomes and safety should precede broad adoption.

Future work should test the toolkit as a pilot implementation project, measuring feasibility, fidelity, time‐to‐analgesia, clinically meaningful pain relief, functional outcomes, patient experience, adverse events, opioid prescribing quality and follow‐up success. Until such data are available, the framework should be viewed as a structured proposal for improving ED pain culture and implementation rather than as a standardised clinical pathway.

## Author Contributions

This position paper was designed by all authors. Michele Domenico Spampinato had a primary role in preparing the manuscript, which was edited by Sossio Serra and Fabio De Iaco. Alessandro Riccardi, Mario Guarino and Claudia Sara Cimmino reviewed the literature.

## Funding

No funding was received for this manuscript.

## Disclosure

All authors have approved the final version of the manuscript and agree to be accountable for all aspects of the work.

## Conflicts of Interest

The authors declare no conflicts of interest.

## Data Availability

Data sharing is not applicable to this article as no datasets were generated or analysed during the current study.
